# Insights into flavor and key influencing factors of Maillard reaction products: A recent update

**DOI:** 10.3389/fnut.2022.973677

**Published:** 2022-09-12

**Authors:** Shuyun Liu, Hanju Sun, Gang Ma, Tao Zhang, Lei Wang, Hui Pei, Xiao Li, Lingyan Gao

**Affiliations:** School of Food and Biological Engineering, Hefei University of Technology, Hefei, China

**Keywords:** Maillard reaction, Maillard reaction products, flavor, flavor mechanism, potential application

## Abstract

During food processing, especially heating, the flavor and color of food change to a great extent due to Maillard reaction (MR). MR is a natural process for improving the flavor in various model systems and food products. Maillard reaction Products (MRPs) serve as ideal materials for the production of diverse flavors, which ultimately improve the flavor or reduce the odor of raw materials. Due to the complexity of the reaction, MR is affected by various factors, such as protein source, hydrolysis conditions, polypeptide molecular weight, temperature, and pH. In the recent years, much emphasis is given on conditional MR that could be used in producing of flavor-enhancing peptides and other compounds to increase the consumer preference and acceptability of processed foods. Recent reviews have highlighted the effects of MR on the functional and biological properties, without elaborating the flavor compounds obtained by the MR. In this review, we have mainly introduced the Maillard reaction-derived flavors (MF), the main substances producing MF, and detection methods. Subsequently, the main factors influencing MF, from the selection of materials (sugar sources, protein sources, enzymatic hydrolysis methods, molecular weights of peptides) to the reaction conditions (temperature, pH), are also described. In addition, the existing adverse effects of MR on the biological properties of protein are also pointed out.

## Highlights

- Implicating the efficiency of MR as natural method for improving flavor in different food model systems and resulting food products.
- Introducing the different Maillard reaction-derived flavors, with an emphasis on the flavor substances.
- A well-controlled MR may be useful in the production of flavor-enhancing peptides and other compounds to increase the consumer preference and acceptability of processed foods.
- The negative effects of Maillard reaction and its solutions are proposed.
- Highlighting the recent application trend of MRPs as flavor agents.

## Introduction

The Maillard reaction (MR) is a non-enzymatic reaction that occurs when the carbonyl group of reducing sugars reacts with the amino group of amino acids, polypeptides, or proteins, resulting in the natural production of Maillard reaction products (MRPs), a class of compounds with a wide range of sensory properties (1). Due to the heteroatoms in amino acids, the resultant scents are heterocyclic compounds with ring structures including an atom of N, O, S, or mixtures of these. In food industry, different flavor and color are produced during the MR, especially as food is heated. This is known not only to alter the food properties (flavor, color, and odor), but also to enhance the functional properties (antioxidant and bacteriostasis) of amino acids, peptides, and proteins (2, 3). Chiang et al. (4) explored how heat treatment affected the volatile profiles of beef bone hydrolysates, and found that MR can be used to alter the flavor characteristics of beef bone hydrolysates as a natural meat flavor product. Song et al. (5) detected the flavor substances in pepper powder and revealed that adding exogenous MR substrate can offer a mechanism to improve BPP flavor quality. In another study, sensory evaluation revealed that soybean protein-derived MRPs have higher umami and caramel traits than soybean protein controls (6). The bitter taste and off-flavor of peptides are regarded as serious challenges to their use in the food industry. The most frequent method for removing or reducing beany flavor components is heating (7). Chen et al. (8) modified the *Cucumaria frondosa* hydrolysate with glucose/xylooligosaccharide by MR which largely increased desirable aroma compounds and reduced off-flavor compounds, improving the overall flavor. These studies implicit the efficiency of MR as natural method for improving flavor in different food model systems and resulting food products.

In MR, flavor relates to peptides of various molecular weights present in amine group substrates. After the reaction, the content of flavor precursors in the resulting products increased significantly, and their umami and strong taste were also improved (9). Until now, over 100 umami peptides (with 2–11 amino acids) responsible for umami or kokumi taste from different sources have been distinguished, such as pea protein hydrolysates (10), sweet potato protein hydrolysates (11), edible mushrooms (12), peanut protein (13), and *Takifugu rubripes* (14). It has been evident that different MR conditions result in different flavor products. Since the open-chain concentrations of sugars and active forms of amino reactants depend on pH (15). Lotfy et al. (16) heated the Quinoa protein hydrolysates at varying initial pH values and increased the pH from 5 to 9 to change the sensory attributes from caramel to burnt-coffee. Furthermore, MR is made up of a chain of complex reactions, in which the sensitivity of each reaction and reactant to temperature are different. At higher temperatures, bitter and umami FAAs were present in higher concentrations (17). Thus, a well-controlled MR may be useful in the production of flavor-enhancing peptides and other compounds to increase the consumer preference and acceptability of processed foods.

Recent reviews on this research area have mainly focused on the MR during processing (18, 19), or their effects on functional and biological properties (2, 20), without elaborating the flavor compounds obtained by the MR. To bridge the knowledge gap, we aim to introduce the MR and different MF, with an emphasis on the flavor substances. The influencing factors, flavor formation mechanism of MRPs, and widely used flavor detection methods are also analyzed. Besides, we highlight the recent application trend of MRPs as flavor agents. The development direction and trend are comprehensively discussed, to provide theoretical support for expanding the existing application standing of MRPs.

## Maillard reaction-derived flavors

**Figure 1:**
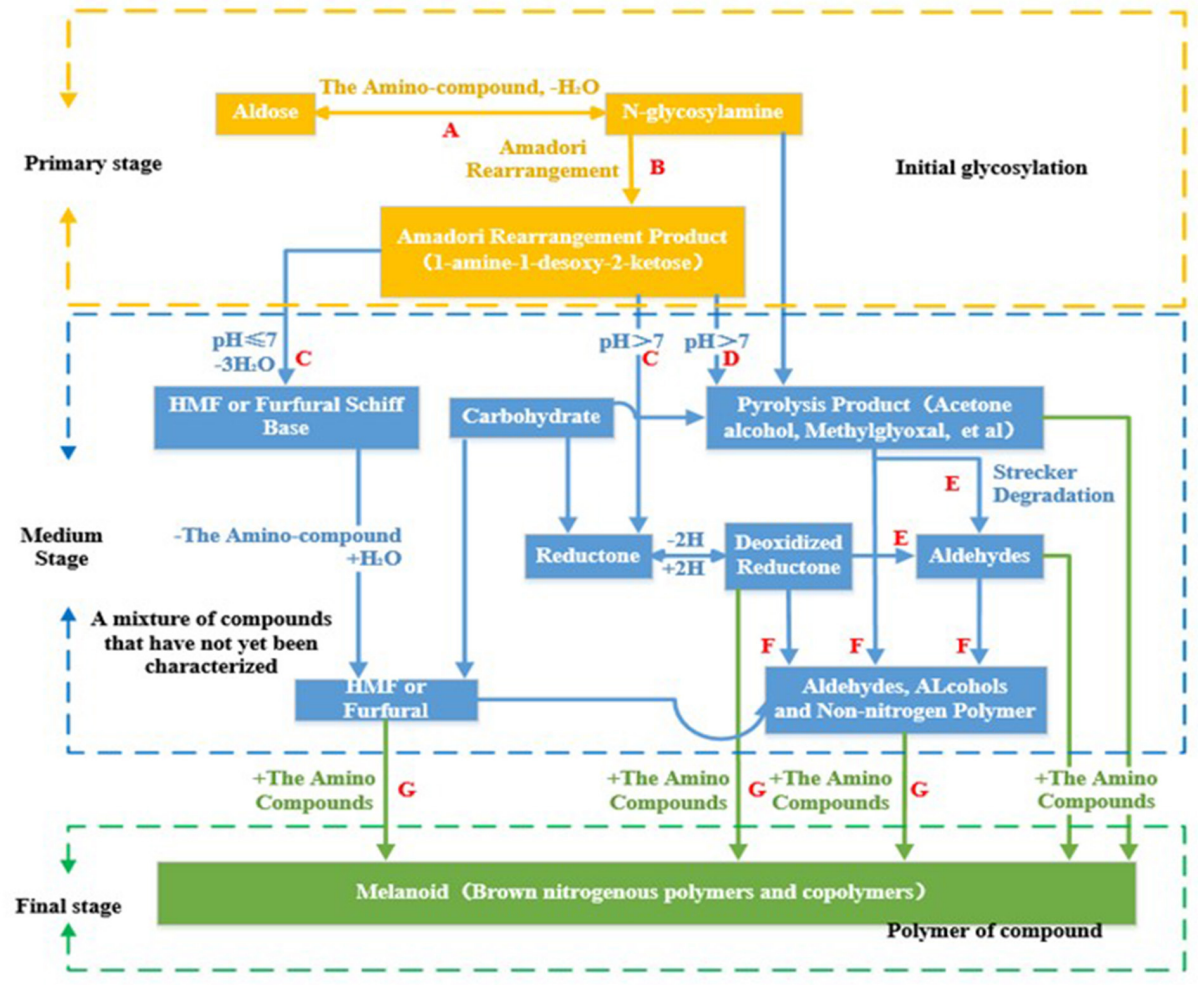
Mechanistic diagram of Maillard reaction.

As the foods containing proteins/peptides and carbohydrates are heated, MR (formation of covalent bonds between carbonyl groups and free amino groups) is a common reaction (17). It started in 1912 with French chemist Louis Camille Maillard, who studied the interaction between glucose and glycine and labeled the resultant dark pigment melanin. Generally, in MR there are three steps, namely primary stage, medium stage, and final stage, as shown in Figure 1.

Amadori products of the primary stage are often used as an indicator of food processing degree (21). In a simple model of cysteine and xylose, relatively stable cyclic 2-3-thiazolidine-4-carboxylic acid (TTCA) and the Amadori rearrangement product of cysteine are formed at the early stage. Gly-Amadori reacts fastest, followed by Cys-Amadori and TTCA. Free glycine accelerates the reaction of TTCA, whereas cysteine inhibits that of Gly-Amadori due to forming relatively stable thiazolidines. Cys-Amadori/Gly has the highest efficiency in developing both meaty flavors and brown products. TTCA/Gly is conducive to yield meaty flavors, whereas Gly-Amadori/Cys is conducive to generate brown products (22). Conclusively, initial formation of initial intermediates/pathways could be regulated for the maximal formation of related flavors.

In the medium stage, the ketone aldehydes and other compounds produced in this process are the flavor precursors and flavor substances of foods. For example, amino ketones could be converted to enol-amines through isomerization. Strecker aldehydes and acids, which have strong aromatic potential, produce different aroma characteristics according to the types of amino acids and act as precursors. Various special aldehydes could be generated by the MR of different α-amino acids and reducing sugars as Strecker degradation (23). Therefore, Strecker degradation is related to browning and aroma production. For example, Val reacts with glucose, chocolate aroma is produced (24). The reaction of Lys and Pro with glucose produces the aroma of toasted bread (25).

The final stage mainly includes dehydrogenation, cyclization, retro-Aldol reaction, isomerization, rearrangement and condensation. Nitrogen-containing dark compounds, namely melanoidins, are formed eventually. In addition, the carbohydrate in MR also is cracked after heating. Ketones, keto alcohols, ketoaldehydes and other crackates react with amino compounds, before the final MRPs are further formed (26).

MF refers to characteristic flavor formed by MR during food processing and storage. The flavor characteristics could be pleasant floral, nutty, caramel, attractive meat, or even spicy (5, 27, 28), depending on the types, composition or reaction pathways of aminos and carbonyls. In 1960, Morton prepared meat flavor by using reducing sugar and cysteine, and applied for the first patent on MF in the UK. Since then, the research on MFs have been continuously progressing. MF is caused by MR that produces compounds, such as aldehydes, ketones, furans, thiophenes, pyrazines, pyrrols, etc. These compounds (MRPs) contribute to the overall flavor perception in foods during the processing. Due to the edible safety of MRPs and the high efficiency of flavor enhancement, the preparation of MFs through thermal reaction has gained an immense focus from researchers engaged in flavor industry and food processing ingredients (8, 16). At present, the key technologies for thermal reaction flavors have been developed, which are composed of adipo-regulated oxidation (28), targeted biocatalysis (28), and temperature-controlled (17).

## Flavor ingredients of MRPs and detection methods

### Flavor ingredients of MRPs

#### Flavor components in MR

Flavor is an important characteristic of MRPs and flavor substances are initially formed in the middle stage of MR. The structure, formation process, and flavor characteristics of some representative flavor substances are summarized in Table 1. Most of MRPs are furans and their derivatives, and the precursors of other condiments (such as thiazole and furan mercaptan). Linoleic acid and other n-6 including 2-pentylfuran, which is a non-carboxylated molecule (28). 1-Octen-3-ol was degraded from lipids and had a mushroom and baking aroma (36), as well as antibacterial efficacy against spoilage and opportunistic microorganisms (37). The MR generated furan compounds, which are a key odor group for hibiscus flowers (38); 3-methylbutanal provides a malty scent, while pyrazines (typical MRPs) contribute a barbeque taste to food, with higher pyrazine levels owing to the possibility of lipid-derived active carbonyl and ammonia reactions (39). MR is known to significantly contribute to baked red pepper powder flavor due to these compounds (5). Meat flavor is characterized by sulfur-containing volatile compounds with sulfury and meaty aromas. Meaty tastes described with high odor in model MR containing cysteine including 2-methyl-3-furanthiol, 2-furfurylthiol, 3-thiophenethiol, 3-mercapto-2-pentanone, bis(2-methyl-3-furyl)disulfide, and 2-methylthiophene (40).

**Table 1:** Flavor characteristics and forming pathway of flavor products.

**Substance**	**System**	**Forming pathway**	**Flavor**	**References**
**Sulfur-containing compounds**				
**Thiophene**				
3-methyl-2-thiophenecarboxaldehyde	a) Peony seed-derived, chicken fat, and L-cysteine b) Camellia seed meal hydrolysates, ribose or xylose or glucose or fructose, and L-cysteine	Derived from thermal degradation of cysteine and carbonyl compounds from lipid oxidation	Cooked meat-like odor	(28, 29)
3-methyl-2-thiophene carboxaldehyde	Bacon and woodchips		Similar flavor of cooked meat and sulfur	(30)
2-methyltetrahydrothiophen-3-one	Tilapia fish head hydrolysate, xylose, and cysteine		Sulfurous, fruity and berry odor notes	(31)
**Thiols**				
2-furfurylthiol	Peony seed-derived, chicken fat, and L-cysteine	\	Strong aroma of baked products, coffee, and fleshy odors	(28)
**Thiazoles**				
2-acetylthiazole	Peony seed-derived, chicken fat, and L-cysteine	Oxidized lipids react with the MRPs	The aroma of nuts, cereals, and popcorn, with a low odor threshold	(28)
Benzothiazole	Potato and glycine		Caramel characteristic, and its threshold is low	(32)
2-Acetylthiazole	Tilapia fish head hydrolysate, xylose, and cysteine	The reciprocity of sulfur-containing amino acids with carbohydrates or carbonyls	Nutty, popcorn, peanut, roasted and hazelnut odor notes	(31)
**Other sulfur-containing compounds**				
Dimethyl trisulfide	Bacon and woodchips	\	An ideal onion-like odor at low concentration and an undesirable sulfur odor at high concentration	(30)
**Nitrogen containing compounds**				
Pyridine	a) Peony seed-derived, chicken fat, and L-cysteine b)Sesame seed hydrolysate, d-xylose, and L-cysteine or L-methionine or thiamine	Trigonelline degradation and MR	Unique roasted, nutty, meaty, earthy, and popcorn-like aroma	(27, 28)
Pyrazine	Potato and glycine	Oxidized lipids react with the MRPs	Sweet	(32)
Pyrrole	Coffee	The carbonyl compounds could either react with amino acids or the sugar degradation products	Nutty, hay-like, and herb aroma	(33)
**Oxygen containing compounds**				
**Ketones**				
3-methyl-1,2-cyclopentanedione	Bacon and woodchips	The degradation of amino acid, oxidation or degradation of unsaturated fatty acids, carbohydrate metabolism, and β-keto acid oxidation	Unique fragrance, fruity, woody aroma, and mushroom-like flavor	(30)
Furanones	Quinoa protein and xylose	Sugar degradation products and can undergo condensation reaction via Maillard reaction	Mainly described to have caramel-like, sweety, nutty and burnt notes	(16)
Cyclopentenones			Caramel and burnt odors	
**Furan**				
2-Methylfuran	Quinoa protein and xylose	Obtain by xylose cyclization	Contributes to the fresh taste of roasted coffee	(16)
2-Pentylfuran	Pork hydrolysate and xylose	Form from the oxidation of linoleic acid	Fruity, floral, buttery, green and beany notes	(34)
**Alcohols**				
Furfuryl alcohol	Bacon and woodchips	\	The warm “burnt” odor and cooked sugar taste	(30)
1-octen-3-ol	Clam hydrolysate	Enzymatic peroxidation of polyunsaturated fatty acids	Strong grassy and fatty odors	(35)
Methanethiol	Tilapia fish head hydrolysate, xylose, and cysteine	The oxidation products of methanethiol derived from the degradation of methionine via the Strecker degradation	Sulfurous, alliaceous and eggy odor notes	(31)
2-furanmethanethiol	Pork hydrolysate and xylose	Furfural reacts with the hydrogen sulfide, which is formed from the cysteine breakdown	A strong and distant “roasted meat” aroma	(34)
**Esters**				
Methyl ester	Bacon and woodchips	The hydroxy acids' intramolecular esterification	Contribute a fruity odor to meat products	(30)
Butanoic acid ethyl ester	Clam hydrolysate	Esterification of an alcohol with a carboxylic acid	Sweet and fruity aroma	(35)
**Aldehydes**				
2-methylbutanal	a) Peony seed-derived, chicken fat, and L-cysteine	Strecker degradation of isoleucine	Malty and chocolate	(28)
Furfural		\	Sweet	
Benzaldehyde		Originates from the oxidation of benzyl alcohol catalyzed by dehydrogenases	Almond-like	
Benzeneacetaldehyde		\	Honey, sweet, and floral	
Hexanal	Bacon and woodchips	Linoleic acid and other unsaturated fatty acid oxidation	A rancid odor in high concentration, a fruity and broth-like odor at low concentration	(30)
Nonanal		Lipid oxidation	A greasy and sweet orange flavor	
Octanal	Potato and glycine	\	Beef aroma	(32)
2-Furfural		Xylose cyclization in the second later stage of the MR	Almond-like aroma	
**Phenols**				
Guaiacol	Bacon and woodchips	Pyrolysis of lignin	A smoky flavor	(30)
Hydroxybenzol		Mainly derived from the thermal pyrolysis of lignin or hemicellulose of woodchips	A pungent, smoky aroma	
**Hydrocarbons**				
α-Pinene and β-pinene	Peony seed-derived, chicken fat, and L-cysteine	\	Unique turpentine aroma	(28)

Furfural, 5-methyl furfural, 2-acetyl furan, maltol, iso-maltol, and other substances have strong caramel and fruit aroma. 2, 5-Dimethyl-4-hydroxy-3-furanone and its 5-methyl homolog have a pleasant caramel taste and roasted pineapple smell. Dicarbonyl compounds (such as butanedione) with buttery aroma are obtained by deoxyglucone rearrangement and dehydration. Aldehydes, such as 3-methyl-butyraldehyde, are formed through Strecker degradation by dicarbonyl compounds, and present a refreshing malt aroma. Pyrazines, which are a kind of flavor compound with pleasant mood and nutty aroma, have attracted much attention from flavor chemistry researchers since the 1960s. They're generally produced by condensation of -aminoketone, which is formed through Strecker degradation. Similarly, pyrrole and its derivatives formed by dehydration and cyclization of 3-deoxyketone with amino compounds are also important flavor substances. When proteins react with methylglyoxal or dihydroxyacetone, 2-acetyl-1-pyrroline with bread aroma is produced. The sulfur-containing flavor substances formed directly relate to sulfur-containing amino acids in the reaction system. Hydrogen sulfide and ammonia are easily produced by cysteine hydrolyzation or Strecker degradation. Similarly, thiophenes are formed by hydrogen sulfide\deoxyglucose after dehydration and oxidation. Recently, MRPs derived from protein hydrolysates/peptides and carbs have been shown in numerous researches to impact the flavor properties of food (8, 28, 41). Table 2 summarizes the flavor attributes of MRPs generated from different protein hydrolysates/peptides and carbs.

**Table 2:** Summary of the recent studies on flavor properties of Maillard reaction products generated from various protein/ protein hydrolysates/peptides and carbohydrate sources.

**Protein/protein hydrolysates/peptides**	**Carbohydrate**	**Maillard reaction conditions**	**Flavor properties**	**References**
Camellia seed meal	Ribose	110°C, 90 min	Meaty and umami taste	(29)
Sesame seed meal	d-xylose	120°C, 2 h	Strong meat flavor	(27)
Takifugu obscurus by-products hydrolysates	Xylose	pH 7.4, 120°C, 2 h	Umami taste, volatile aroma, and overall acceptance ascension, bitterness and fishy smell reduce	(42)
*C. frondosa* hydrolysate	Xylooligosaccharide	115°C, 20 min	Stronger sweety, baked, and caramel notes, weaker seafood and fishy odors	(8)
Pork hydrolysate	Xylose	pH 4.5, 100°C, 1 h	Roasted and sweety taste	(34)
Large-leaf yellow tea	\	145–155°C, 3.5 h	Strong roasted, nutty, woody odors and weak fatty, fruity odors	(43)
Grass carp hydrolysate	Glucose	120 °C, 1 h	Caramel and bitterness reduce, and overall acceptance ascension	(44)
Clam (Aloididae aloidi) hydrolysate	\	70°C, 10 min	Stronger pleasant flavors, less green, grassy and fishy odors	(35)
Agaricus bisporus mushrooms	\	176.7°C, 4–6 min	Dark meat, roasted, and fried notes, and portobello increased. Woody, and earthy notes decreased.	(30)
Oyster meat hydrolysate	Glucose	pH 7.0, 115°C, 35 min	Overall acceptance ascension	(45)

**Figure 2:**
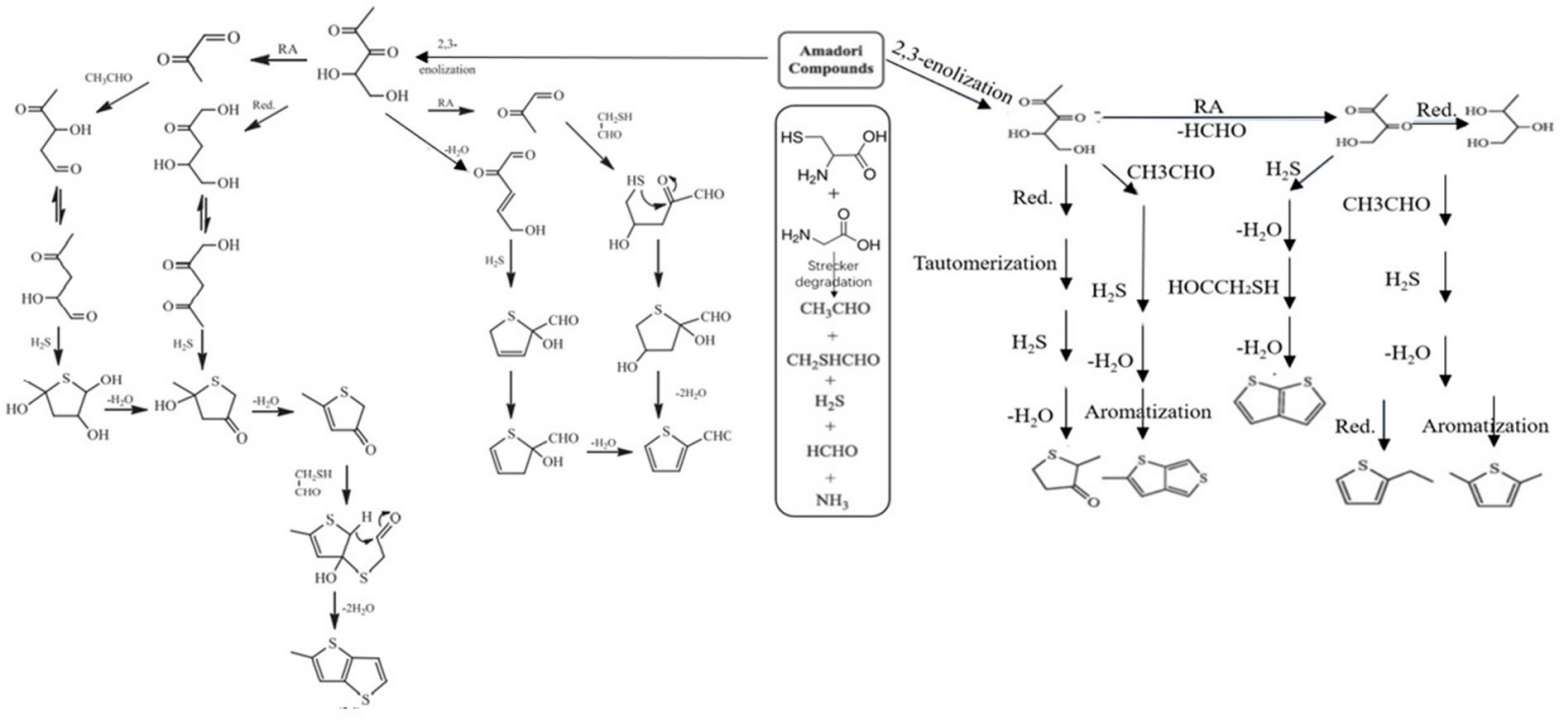
Possible formation pathways for compounds in the Gly-Cys reaction systems (Red., reduction reaction; RA, retro-aldol reaction) (40).

In the heated cysteine-xylic-glycine system, the possible processes of different compounds are summarized in Figure 2. 2-Methyltetrahydrothiophene-3-ketone could be formed from 1-deoxypentanone through reduction. Furfural may be generated from 3-deoxypentosone *via* cyclization and dehydration, while 2-furfurylthiol could be formed from the reaction of ffurfural and H_2_S. Besides, 2-methylfuran could be formed after the reduction of furfural. After 2,3-pentadione reacting with H_2_S, 3-mercapto-2-pentanone may be generated. Analogously, after cyclization and dehydration with hydrogen sulfide, 3-thiophene mercaptan is formed. Subsequently, 2-ethylthiophene is formed through dehydration and reduction. As aromatization occurs, 2,5-dimethylthiophene is formed (40).

#### Flavor components in foods associated with MR

In heat processed foods, such as coffee, cocoa, bread, peanuts, pork, beef, chicken, fish and potatoes, most aromatic substances are produced by MR. Over 400 volatile compounds including pyrazines, thiazoles, oxazoles, pyrrole derivatives, furans, and pyridine derivatives have been identified in roasted cocoa. Furthermore, the pattern of alkyl pyrazines generation depends on oligopeptides and free amino acids presented in the particular foods before roasting (46). Kocadagli et al. (32) selected precursors to add to potato dough before baking for improving the baked flavor of low-acrylamide potato products. Due to the reactive complexities of tea, noticeable changes such as pigment destruction, oxidative polymerization of catechin, and oxidative degradation of amino acids have been recorded following high-temperature esterification and long-term storage. Compared with the roasted spring and autumn tea leaves, the color, stewing aroma, taste and chemical composition of pan-fired spring tea leaves changed to least, indicating that baking enhanced the flavor stability of tea drinks (47).

In addition to improving the food taste, MR is also used to improve the food smell. For example, shellfish hydrolysates usually produce strong unpleasant odors (such as fishy odors), which is the key reason for their limited application (47). After MR, the number of volatile components detected in the enzymatic hydrolysate of clams was changed insignificantly. Nevertheless, there were differences in the types, and the contents of aldehydes, ketones and esters were also increased. After enzymatic hydrolysis, MR eliminated the unpleasant smell of oyster, and a pleasant smell of milk, nut and meat was generated at the same time (48).

### Extraction and detection methods of MRPs

Extraction and analysis are important technical strategies to study the food flavor components. Flavor components are mainly composed of many volatile substances with complex structures and different chemical properties. They act on olfactory organs to transmit information, and their contents are usually below the nanogram level. Therefore, it is relatively difficult to analyze their chemical composition by conventional chemical methods. Analysis of the chemical composition usually includes separation, enrichment, desorption, and identification of volatile components. All the processes should avoid oxidation, thermal degradation and other biochemical reactions, which are the prerequisites for pretreatment of volatile compounds. Common methods for samples pretreatments include solvent extraction (SE), simultaneous distillation extraction (SDE), headspace extraction (HS), solvent assisted flavor evaporation (SAFE), super-critical fluid extraction (SFE), purge and trap method (PT), solid phase microextraction (SPME), etc. The commonly used analytical instruments for the identification of volatile aroma compounds in food systems include electronic nose (E-nose), gas chromatography (GC), and high-performance liquid chromatography (HPLC).

E-nose is a rapid detection system suitable for detecting large number of samples, without the need to separate the volatiles. Using E-nose as a human sensory simulator, sensory evaluation could be conducted conveniently and sensitively. Currently, E-nose is used to analyze the overall level of aroma components for MR conditions optimization (49). However, the electronic nose cannot detect the quantity and content of each substance in MRPs. An useful analytical technique for identifying MRPs is HPLC or anion-exchange chromatography in combination with a diode array detector (DAD) and an evaporative light scattering detector (ELSD) (50). The majority of these approaches, nonetheless, did not succeed in clearly identifying each MRP with good peak shape and resolution. The high polarity of MRPs, which results in early elution with many compounds in the reversed phase (RP) column and substantial retention in the normal phase (NP) column, is attributed as one major cause. Satisfactory separation of polar substances is now possible thanks to the recent invention of the hydrophilic interaction liquid chromatography (HILIC) column, which strikes a fair compromise between RP and NP columns. Its use concurrently separates the amino acids glycine, diglycine, and triglycine as well as the associated MR intermediate (51).

These analytical techniques have limitations, such as low selectivity, especially when compared to cutting-edge analytical techniques utilizing mass spectrometry (MS) and nuclear magnetic resonance (NMR) spectroscopy (52). For the analysis of both tiny and large organic compounds, MS is regarded as one of the fundamental methods (53). Sensory assessment, GC-MS and E-nose were used to investigate the flavor differences of five sections of the Chinese blanched chicken (CBC): skin, breast, thigh, head, and butt. This examination of several aspects of CBC might give guidance on how to cater consumers' preferences and forecast the quality changes that may occur. Additionally, using a combination of GC-MS and E-nose, the volatile and taste components of foods (such as golden pompano filets, roasted coffee beans, apple fruits, and so on) were evaluated to gain complete information on flavor profiles. Nowadays, GC-MS is a popular approach for analyzing aroma that has been widely applied in meat science. For example, the investigation of volatile profiles of fermented sausages, dry-cured hams, and bacons (54, 55). It provides an accurate approach to qualitatively and quantitatively analyze the volatile and semi-volatile compounds (56). GC-MS, on the other hand, requires extra derivatization and is not appropriate for large-scale detection (57).

MS is a standard mass detector that detects ionized chemicals and delivers mass spectra and compound structures at each location. MS/MS or even MS^3^ approaches improved selectivity and structure identification by analyzing each ion fragment of a molecular ion (58). In a recent investigation, the standards of glycine-glucose and proline-glucose-Schiff bases, Amadori, and Heyns compounds were well separated by ESI-MS/MS (59). Additionally, techniques utilizing LC-MS/MS and stable isotope dilution assay were proposed (60). The identification of tiny molecules like ARPs in a complex matrix becomes feasible and reliable, which is favorable and has a wide range of applications, despite certain hurdles brought by the high cost of MS detection and onerous technique.

A key component of MS approach is the MS Library; flavor analysis, for instance, benefits from the NIST library (61). A dedicated library for Maillard reaction chemicals does not exist, though. On the basis of ion fragments, researchers must infer the molecule and/or further confirmed by NMR. Only a few attempts have been made to create tiny libraries of sulfur-methyl thioesters so far, and they haven't been put into too much use. Therefore, the creation of a thorough library of MRPs would be a significant addition.

## Influencing factors of MF

### Influencing factors of MF

The sort of amino acids and reducing sugars, as well as the reaction circumstances (such as temperature, time and pH), play a big role in flavor development *via* MR. The sugar has only a limited influence on flavor character, while the amino acid has a major impact. Rather than altering the flavor character, sugars have a significant impact on the reaction rate. The influence of different influencing factors of MRPs is showed in Table 3.

**Table 3:** The influence of different influencing factors of Maillard reaction products (MRPs).

**System**	**Maillard reaction conditions**					**Influencing factor**	**Observations**	**References**
	**pH**	**Time (min)**	**Temperature (°C)**	**Protein: sugar ratio**	**Heating method**			
Camellia seed meal hydrolysates and ribose/xylose/glucose/fructose and L-cysteine	\	90	110	2:1	Wet	Sugar species	Ribose-MRPs and Xylose-MRPs were more common in meat and umami flavors than other MRPs. There was no greater diversity between MRPs in taste, saltiness and continuity strength.	(29)
Sesame seed meal hydrolysates and d-xylose and l-cysteine/l-methionine/thiamine	7.5	120	120	10:3	Wet	Amino acid species	The MRPs with cysteine showed the strongest flavor. The MRPs with thiamine showed the highest meat flavor, followed by Cys-MRPs. All of the MRPs have lower bitterness and stronger caramel flavor.	(27)
Beef bone hydrolysates and d-ribose	6.5	10	113	1:0.068	Wet	Protease species	When comparing Flavourzyme -MRP in single hydrolysis treatment with both Protamex + Flavourzyme -MRP and Bromelain + Flavourzyme -MRP in simultaneous hydrolysis treatment, there were no significant differences on those major volatile compounds for the three MRPs. This showed that Flavourzyme^®^ is effective in generating major volatile compounds during MR, without the need for addition of other enzymes.	(4)
Rapeseed peptide, d-xylose, and L-cysteine	7.4	120	80/100/120/140	5:1.4	Wet	Reaction temperature	The sample reacted at 80°C had a significant effect on mercaptan content, the 100°C sample showed a significant effect on aliphatic sulfur compounds, indole, indazole, esters and Tyr, showing more umamy and saltier taste. While the 140°C sample was rich in nitrogen oxides. They are closely related to thiophene, furan, pyrrole, pyrimidine, pyrazole, pyran, aldehydes, Thr, Met and Leu.	(17)
Duck legs	\	45	85/95/121	\	Wet	Reaction temperature	Thermal treatment caused further Maillard reaction in the water-boiled salted duck, although the degree of this change was small	(62)
Pork, d-xylose, and L-cysteine	\	5 min at 120 and 90°C for 25 min		200:1	Dry	Thermal way	High-temperature stewed pork had the significant effect on the formation of furans, N- and S-containing compounds, the processing technology of traditional stewed pork was more sensitive to aromatic compounds.	(63)

#### Reducing sugar

The MR refers to the reaction between an amino group (e.g., amino acids) and a carbonyl group (e.g., reducing sugars), a non-enzymatic modification of proteins with reducing sugars. Therefore, sugars were a key participant of MR, and the content and structure of reducing sugars have an important effect on the MR and the structure and flavor of MRPs. The level of the reducing sugars inside the beans has a potential impact on coffee aroma, flavor and color during roasting process (64). In the searing-cooked steak, the reducing sugar was lower and MRPs were higher than oven-cooked steak, which had higher scores for overall flavor and roasted meat flavor (65). Nevertheless, one of the limitations of these studies is that they only studied the effect of the total reducing sugar content in the system on the flavor produced by the Maillard reaction. It is not explained which reducing sugar plays the key role. Deng et al. (66) found reducing sugars such as glucose and ribose could react with α-amino acids *via* the Maillard reaction and promote the formation of the cooked meat aroma compound. Furan is a necessary intermediate in some chemical systems and composed of pentose and generated by Amadori intermediate 1, 2-enolization. In the Maillard reaction with xylose, the content of furfural and meat related volatile flavor substances was higher than that with ribose and glucose (29). Furthermore, the addition of reducing sugars could induce protein glycation and facilitate the solubilization of myosin, which in turn might increase the water holding capacity of meat (66). However, only the sensory evaluation of the products after adding different sugars is not enough. According to the current research, it can only be confirmed that the addition of reducing sugar is beneficial to the formation of flavor, and xylose is relatively widely used. These studies would be more relevant if the effects of sugars on the flavor orientation produced by the MR were explored more broadly.

#### Source of proteins

Proteins of different sources react with sugars and further produce different flavor of MRPs. In recent years, more and more vegetable proteins or peptides have been used in the production of flavor enhancers by MR. Soybean peptides have been used to generate a range of flavors, including umami, sweet, salty, and meaty, as a potential flavor precursor (67). MRPs were made utilizing a xylose and soybean peptide method after heating at 100–140°C and pH 7.6 for 2 h. MRPs with cysteine addition presented the minimal bitterness, after heating at 140°C, and the highest umami and saltiness at 100°C (68). Not only soybeans, but other plant-derived proteins are also reported to change the flavor through MR. In umami soup, MRPs of flaxseed protein hydrolysates could enhance the mouthfulness and continuity (68). And hydrolysates or extracts from meat have also been employed to improve the meat-like flavors of food products. However, the main drawback of these studies is that the resulting Maillard reaction products have no clear directivity (chicken, beef, or fish flavor).

To further enhance the flavor, Kang et al. (69) reported that after addition of xylose and L-cysteine to beef hydrolysate, kokumi, meaty, umami, umami-enhancing and kokumi-enhancing capacity were improved. However, In comparison to meat hydrolysates, meat extracts have poor flavor and aromatic qualities. Enzymatic hydrolysis and MR could turn animal bone extracts into taste components. To solve this problem, ribose was utilized to investigate MRPs generated from beef bone hydrolysates. Enzymatic hydrolysis and heat treatment are reported to promote the production of meat characteristic flavor substance (2-methylpyrazine, dimethyl disulphide, and dimethyl trisulphide) and inhibit the burning taste and formation of bitter substance (2-furanmethanol) (4). Similar results were also observed in swine lard and goat by-product protein hydrolysates, in which aroma compounds were conducive to an increase in the hydrolysis process (70). By markedly increasing the contents of pyrazine and sulfur compounds in the hydrolysate, Flavourzyme could improve the flavor of chicken bone extracts. MR significantly decreased the bitter taste and increased the flavor acceptability of hydrolyzed chicken bone extracts by using Protamex^®^ and Flavourzyme^®^ in a sequential hydrolysis procedure (71). Using chicken as a source of proteins, after thermal treatment with xylose, meat flavor was generated, and the thick, kokumi and freshness of the freshness solution were significantly enhanced (72). Moreover, in the recent study, strong barbecue beef flavor was generated with bone protein hydrolysate (73, 74). To improve the surplus value of fish, heating the low-valued fish hydrolysates with meat hydrolysates, xylose and other additives were optimal for sauce flavor synthesis, in which meaty smell was strengthened and fishy odor was reduced. For example, after xylose reacted with the hydrolysate of shrimp, a product with rich seafood taste and freshness was obtained. After xylose was heated with the polypeptide hydrolysis of shrimp, the resulting MRPs had a rich seafood taste and freshness (75). The hydrolysate of fermented Tilapia fish head could develop a flavor concentrate (31). Overall, a convergence between meat proteins and the flavors of their MRPs was showed, which indicated that we could target the raw materials for MR. However, these studies mainly focused on meat flavor and lacked systematic research on other flavors.

#### Hydrolysis conditions of raw material

The specific substances with flavor characteristics in MR are affected by specific amino acids, peptides, and proteins. The hydrolyzed products obtained by different peptidases contain different peptide spectral and free amino acids, its MRPs also have different sensory properties. *A. Melleus* contains a large number of peptides below 1,355 Da (probably corresponding to dipeptides or aromatic amino acids). In the meantime, under the hydrolysis of some proteases such as chymotrypsin or pepsin, the proteins were cleaved specifically to produce peptides that had a c-terminal amino acid (76). Grossmann et al. (77) found that the bitter and umami taste of the cricket and mealworm protein samples had an increasing tendency after enzymatic hydrolyses, which coule be clarified by the liberation of amino acids. However, not all proteases play decisive roles in flavor. Five kinds of bovine bone hydrolysates were genarated by single (P-Protamex^®^, B-bromelain, F-Flavourzyme^®^) and simultaneous (P+F and B+F) enzymatic hydrolysis, then were heated at 113°C to form MRPs. The proportion of most volatile compounds in P+F-MRPs and B+F-MRPs differed insignificantly as Flavourzyme^®^ reacted with Protamex^®^ or bromelain simultaneously. Volatile components (such as 2-methylpyrazine, dimethyl disulfide and dimethyl trisulfide) were detected in B-MRPs after heating, and did not appear in P/F-MRPs. They contributed to baking, sulfur, and meat flavor, but the difference was not significant. Based on it, comparing the single hydrolysis of F-MRPs with the simultaneous hydrolysis of P+F-MRPs and B+F-MRPs, there was no remarkable difference in the main volatile compounds of the three MRPs. This suggested that only addition of Flavournzyme^®^ was effective in producing major aroma compounds during MR (4). Various studies have fully demonstrated that protease specific cleavage is one of the means to improve the sensory quality of MRPs. However, the mechanism of flavor enhancement by enzymatic hydrolysis was not considered. It would be more meaningful to further study the characteristic precursor peptides that generate flavor substances in MR. Other enzymes besides proteases also contribute to the formation of flavor in MR. For example, meat-like flavors can be created by myrosinase and lipoxygenase in the two-step brom of Brassica proteins (78). Reducing sugars produced by enzymatic hydrolysis of starch play a role in MR, which can alter the production of taste components directly or indirectly (79). The content and reactivity of MR substrate were increased by enzymatic hydrolysis, which provided the prerequisite for the production of flavor. After the selection of raw materials, the type of enzyme can be selected according to the requirements of the reaction and material characteristics. When dealing with substances with high protein content, proteases can be selected for enzymatic digestion to obtain more highly active peptides. As raw materials with high carbohydrate content were utilized, amylase and glycosidase could be used to increase the content of reducing sugar that can participate in MR.

#### Molecular weight of peptides

Peptides have been considered to be very critical taste active ingredients in foods. Peptides of molecular weight ranging from 1 to 5 kDa have been found to strongly influence the flavor enhancing attributes (umami, continuity, and mouthfulness), and they are called Maillard peptides; which mainly contribute to the kokumi taste (41, 80). The scent of chicken was provided by a peptide fraction of 2–5 kDa in chicken enzymatic hydrolysate. Meanwhile, in MRPs of chicken enzymatic hydrolysate, a peptide fraction < 500 Da contributed to the roasted scent of chicken, a peptide fraction > 1 kDa formed by cross-linking the peptide fraction 500 Da contributed a kokumi flavor, and a peptide fraction > 3 kDa imparted a bitter taste (81). Low molecular weight (LMW) peptides (<1 kDa) generated in meat during chilled conditioning can act as flavor precursors in the MR with a potential contribution to key volatile organic compound (VOC). The majority of nitrogen-containing volatiles, pyrazines and pyridines, dominated the carnosine mixture, while sulfur-containing VOCs dominated the GSH and Cys Gly peptide mixtures. The peptides with LMW (<1 kDa) are mainly the products of degradation of High molecular weight (HMW) peptides, which are considered as the main contributor of the formation of pyrazines and 2-furfurylpyrrole due to the higher reactivity of the amidogen in these compounds. HMW peptides are the products of cross-linking and polymerization of LMW peptides, which may be formed during the MR. However, peptides with a molecular weight of 128–1,000 Da were found to be primarily responsible for the meat-like flavor, as well as influencing other sensory characteristics (68). Various studies have well demonstrated that peptides with molecular weights < 5 kDa are more suitable, and HMW peptides tend to bring bitter and other odors. Thus, specific enzymatic hydrolysis or ultrafiltration can be used to increase the content of LMW in the reaction system, to increase the consumer preference and acceptability of processed foods.

#### Reaction conditions of MR

Since the open-chain concentrations of sugars and active forms of amino reactants depend on pH, the parameters significantly influenced the reaction rate and formation of MRPs (15). Firstly, high pH provided favorable conditions for the molecular rearrangement of sugars, and could promote the occurrence of nucleophilic addition reaction (82). Secondly, Amadori compounds are prone to form 1, 2-enolization at pH 8, and tend to react with 2, 3-enolization at pH 9.7. Thirdly, increasing initial reaction pH was beneficial to caramelization, but it had little effect in the range of 6.7–8.0 (83). Therefore, pH might alter the route of MR, causing in variations in the types and contents of flavor compounds in products. During the MR, the consumption of amino groups, the degradation of sugars, free amino acids (FAAs), and peptides facilitated the formation of acidic compounds. For example, after the addition of cysteine to the soybean peptide and D-xylose system, terminal pH was decreased, because cysteine accelerated the formation of formic acid and acetic acid (68). For the first time, Lotfy et al. (16) used enzymatically hydrolyzed quinoa protein as a major precursor for producing thermal process flavorings. By increasing pH from 5 to 9, the odor sensory attributes of the generated process flavor changed from caramel to burnt-coffee. This was due to the fact that low pH favored the production of furans, while high pH preferred the formation of pyrazines. However, pH does not have a significant effect on MRPs in all the cases. Li and Liu (34) discovered that the volatile profiles of heat-treated pork hydrolysate might not unaffected by pH. Volatile compounds such as furfural and furans were formed in similar amounts in heated samples with varying pH values.

MR is composed of a series of complex reactions, in which the sensitivity of each reaction and reactant to temperature are different. Therefore, temperature is also known to directly or indirectly influence the reaction degree and product composition. The MR rate increased significantly with the increase of temperature. For every 10°C increase of temperature, the reaction rate increases 3–5 times. At temperature < 110°C, the system of polypeptide and xylose was given priority to cross-linking reaction. At temperature > 110°C, a series of small molecular compounds of degradation were formed in the peptides system. These small molecular compounds accelerate the transition from pyrazine, sulfur-containing compounds and pyrrole to volatile compounds. Furans are sugar-derived compounds that are commonly generated under high thermal treatment (84). The concentration of furans in the soybean peptides-xylose system rose, as the temperature climbed from 80 to 140°C, demonstrating that furans were formed as a result of severe thermal treatment (68). Similarly, as xylose and chicken hydrolytic peptides were heated at 80–100°C for 60–90 min, the formation of umami-flavor and thick-flavor substances was conducive. While pyrazine, furan and pyridine were heated at high temperature (100–140°C) for 30–60 min, the formation of volatile compounds such as with barbecue and meat flavor was conducive (81). On one hand, after high-temperature treatment, ketones and phenols may be converted into intermediates for heterocyclic compounds or volatilized, and esters could be hydrolyzed into acids and alcohols. On the other hand, Protein hydrolysis may produce more amino acids, peptides, and small molecule compounds to accelerate the MR process. Lower heating temperature (80–100°C) with cysteine added was beneficial to forming umami FAAs, while bitter FAAs were likely formed at temperature ranging from 100 to 120°C which was consistent with the recent study for stewed pork with the high-temperature processing methods (63). However, excessively high temperature would not only generate pungent odor substances such as thiazole, but also destroy the enzyme binding sites of polypeptides and generate some cellulose analogs. The analogs are not easy to be decomposed and absorbed by the human body, thus reducing the nutritional values of food. In addition, at temperature > 110°C, some toxic substances (such as acrylamide and AGEs) are formed, which have certain toxic effects on human body.

### MF assistive technology

Recently, new emerging technologies, including pulsed electric field, microwave radiation, high-pressure homogenization and high pressure, have been applied to promote the Maillard reaction with less processing time (35). MR could be significantly accelerated, since ultrasound provided more energy during proteins grafting (85). Furthermore, ultrasonic treatment has the potential to alter the secondary and tertiary structures of proteins, reducing glycation time and increasing the functional properties of conjugates (86). Under ultrasound treatment, the reactivity between proteins and sugars was extremely low. The protein-dense quaternary and tertiary structures protect the active amino groups, and make the protein graft difficult. In contrast, this process usually requires higher temperature and longer time (usually several days) without ultrasound treatment. As an alternative strategy, ultrasound is an appropriate choice for promoting protein saccharification. MRPs had a higher content of conjugated amino acids after ultrasonic pretreatment, indicating that ultrasonic pretreatment of soybean protein/sugar mixture before heating accelerated the reaction (87). Ultrasound assisting MR has been a promising approach for improving the functional properties of mung bean protein isolates with glucose (88). It could accelerate the MR rate, and improve the content of sulfur containing volatile flavor compounds and antioxidant properties of the products (89). Many studies have found that ultrasonic pretreatment of proteins before enzymolysis hydrolyzed the protein into smaller fragments, resulting in a considerable increase in reaction rate (90, 91). Comparing energy aggregating ultrasound (EGU) and energy dispersing ultrasound (EDU) pretreatments, the taste and overall acceptability of MRPs were improved. Due to the low intensity and uniformity of the ultrasonic waves generated by the EDU, the protein molecule distribution was more uniform, which increased the irregular curvature and surface hydrophobicity of the protein. It also resulted in the increase of the content of peptides and amino acids in the hydrolyzed protein as well as the ratio of small molecule peptides to amino acids, thus promoting the MR between hydrolyzed protein and glucose (11).

Traditional production methods of black garlic could not meet the industrial demand because of long-time, high production cost, high-energy consumption, and uneven product quality. The most important chemical reaction in the production of black garlic is MR, and the major substrates are reducing sugars and amino acids (92). The reducing sugars produced by the degradation of polysaccharides and enzymes are important parts of MR, but the enzymes and substrates are usually isolated at different locations in intact cells. The destruction of cellular structures by high-pressure pretreatment affected the intracellular environment, and especially accelerated the degradation of fructan. MR substrates accumulated rapidly in the early stage of processing, which led to the accelerated arrival of the reducing sugar equilibrium point (RSBP) during the heat treatment. High pressure reduced the production time of black garlic from 24 to 15 d, and the taste was also improved (93).

A new non-thermal food processing method is pulse electric field (PEF). Electric field strength, polarity, pulse time, pulse count, and pulse shape are the basic PEF process parameters. PEF processing has so far been widely employed in the extraction of physiologically active compounds, alteration of biomacromolecules, augmentation of chemical processes, and other aspects (94). PEF processing has the ability to change the microstructure and macromolecular interactions of biomacromolecules while reducing the overall processing time with great efficiency when compared to conventional heating techniques. PEF is particularly vital for improving interactions between proteins and polysaccharides (95). Researchers claim that PEF processing might boost the carbon backbone's internal energy fluctuation, enhance mass transfer, and lower the chemical reaction's activation energy (94). The molecular chains of proteins would be attacked by the free radicals produced by electrolysis or electrochemical reactions when the protein-polysaccharide mixed solution is subjected to the PEF processing, leading to the partial-unfolding of proteins' secondary structure and improved protein-water interactions. Additionally, several previously hidden cationic, surface-free sulfhydryl, and hydrophobic groups on proteins are revealed (95). The delivered electric energy simultaneously induces the depolymerization and breakdown of carbohydrate chains. These modifications facilitate the binding of various reactants and increase the likelihood of collisions between reactive molecules. When the free -NH_2_ of proteins links with the -C = O of polysaccharides, covalent interactions take place. By-products of Rainbow Trout (Oncorhynchus mykiss), Dover Sole (Dover Sole) as well as other animal protein (96) and non-dairy plant-based Beverages (97) have been demonstrated to taste better and be more widely accepted when subjected to pulsed electric fields. However, there is presently no large-scale PEF technology available to generate MRPs for the food business. To fully comprehend the interaction between the critical intensity and the thermal consequences of PEF processing, more study is required.

## Negative effects

It is widely known that the average human diet contains large amounts of MRPs. A certain degree of browning plays an important role in developing the sensory properties (such as color, aroma, and flavor) which are needed in certain foods. For example, many baked goods, such as cookies and bread, have desired brown color, giving the consumer an impression of high quality. However, excessive MR might also undermine the sensory, flavor and nutritional values of foods, and even produce anti-nutritional or toxic substances such as acrylamide, heterocyclic amine, and advanced glycation final.

MR might affect the color of agricultural products, causing economic losses. The color deterioration of shrimp was noticed due to the multiple effects of lipid oxidation, phenol oxidation, MR, and astaxanthin degradation (98). Pyrazine and 5-hydroxymethyl furfural (5-HMF) were increased in a time-dependent way. Similarly, the internal browning depreciated the economic value of sweet potatoes due to the generation levels of reactive oxygen species, active polyphenols in the browning area were higher than those in the normal area. Intracellular glucose accumulation initiated the MR and led to ROS aggregation (99).

Moreover, MR played a key role in browning during the instant controlled pressure drop-assisted hot air drying of the apple slices which reduced the sensory quality of the slices (100). As one of the carcinogenic MRPs, acrylamide should be prevented from generation in food systems containing reducing sugars and asparagine. To control the acryl amide generation, ultrasound-MR could be used to accelerate MR in the asparagine-glucose model system (100).

The stability of flavor and flavorings is receiving more attention these days. Flavor loss and deterioration are significant restrictions during food processing and storage, particularly at temperatures above room temperature. Because of their instability (such as pyranone and unsaturated diones), MRPs are extremely prone to flavor loss during storage and processing (101). The flavor quality and customer acceptance of food were both lowered as a result of the loss of favorable fragrance components (such as acetaldehyde, butanal, and furfural). The contents of taste components in MRPs were drastically reduced after storage, and the solution's clarity deteriorated. The stability and transparency of Maillard reaction intermediates as a spice precursor also make them a potential substitute for MRPs (102).

In addition to the food system, MR also occurs in the human body, and could pose a health risk. It is known that the superoxides product might oxidize biological macromolecules (such as lipids and proteins) in each stage of MR in the medical field. Diabetes is related to advanced glycation end products (AGEs), as a result of the fact that MR is triggered by an increase in blood sugar level (103). Furthermore, AGEs accumulation in follicles by activating ATF4 in the follicular microenvironment, which triggers inflammation and reduces oocyte competence (104). In a concentration-dependent manner, AGEs significantly accelerated BMSC senescence, induced mitochondrial dysfunction, and blocked mitophagy (43). Therefore, it is necessary to imply intervention measures to control MR under these situations.

## Application of MRPs as flavor enhancers

Table 4 showed the latest studies on the role of the Maillard reaction in the production of flavor. By studying the taste characteristics of 1–5 kDa peptides MRPs, the Maillard peptide could enhance the umami and continuity of umami solution and consommé soup (43). Heo et al. (111) investigated on the molecular and sensory properties of y-glutamyl peptides, which are key factors to the Kokumi taste of edible beans (*Phaseolus vulgaris* L.). As added to a savory matrix like sodium chloride and monosodium glutamate solutions or chicken broth, the y-glutamyl peptides greatly improved mouthfullness, complexity, and shelf life. Similarly, the Maillard-peptide and the taste enhancer from long-term ripening of miso (soybean paste) were deemed essential substances, suggesting the characteristic flavor (mouthfulness and continuity) of long-ripened miso. Purified di-, tri-, tetra-, and other short peptides have also been extensively studied in MR systems to provide various flavor and taste characteristics. According to previous study, the cross-linking of peptides in the thermal reaction process played an important role in the aroma and mellow feeling. MR study of rapeseed peptides, cysteine and Xylose showed that high temperature reduced the bitter value of the product, and low temperature promoted the salty and umami-tasting (85). Kang et al. (69) reported that interaction between enzymatic hydrolysis and MR improved the quality of beef flavor by regulating the formation of characteristic aroma precursors. In addition, the possible pathway and mechanism of the characteristic flavor of natural meat were further clarified, forming a new technology for the preparation of high-fidelity MF.

**Table 4:** The latest studies on the role of the Maillard reaction in the production of flavor.

**System**	**Application**	**Effect**	**References**
Sesame seed meal protein hydrolysate (SSH)-Cys/Thi	Low sodium seasoning salts	The Cys-MRPs salt had the smallest angle of repose, the highest bulk density, and the highest sensory score. The seasoning salt with SSH-MRPs had appreciable hygroscopicity and thermal stability. The seasoning salt with Thi-MRPs had the highest solubility.	(105)
Glutathione-xylose	Meaty food	Significantly improve and stabilize the flavor quality of the meaty food	(106)
Pea protein hydrolysates- Arabinose	Seasoning salt	Greater umami-enhancing and saltiness enhancement abilities	(10)
Beer yeast, chicken meal, chicken liver, and soybean meal- xylose	Dog foods	Improve palatability of dog food	(107)
Oyster meat hydrolysate- glucose	Oyster meat	The flavor and antioxidative activity of oyster meat hydrolysate MRPs was significantly improved	(45)
Characterized flavor peptides from beef enzymatic hydrolysates- xylose	Beef broth	Stronger meaty delicious flavor and flavor enhancement ability	(108)
Wheat bran	Wheat flavor additive	Added to flour products to enhance flavor and acceptability	(109)
Bovine meat/heart hydrolysates -Glucosamine	Savory applications	Higher in umami, and thereby good candidates for savory applications	(110)
Porcine plasma hydrolysate-Glucosamine	Meat products where liver flavor is desirable	Higher in umami and liver flavor	
Peony seed meal protein hydrolysates- xylose-cysteine-chicken fat	Thermal processing meat flavor	Improve meat flavor and bioactivity	(28)

In the recent years, the promising industrial avenues for MFs have a good potential in China due to the rich flavor, however, there is still scope to develop their application. In view of this characteristic, strategies can be applied to obtain MFs directly by chemical synthesis after analyzing the structural characteristics of key flavor compounds. Zhai et al. (112) synthesized 2, 5-dimethylpyrazine, 2,3, 5-trimethylpyrazine and benzyl alcohol by palladium-catalyzed synthesis of 2-enylpyrazine oxides. Kocadagli et al. (32) also prepared 2-acetyl-1-pyrroline, 6-acetyl-1,2,3,4-tetrahydropyridine and 5-acetyl-2,3-dihydro4H-1,4-thiazide to enhance the flavor characteristics of baked foods. However, resulting MRPs were often unstable and highly volatile, especially at higher temperatures (101). In addition, flavor substances with active properties are often derived into other substances through peroxidation, polymerization and condensation, thus changing the overall flavor profile. Among the MRPs, pyrazine is regarded as the most vulnerable key aroma substances. Followed by pyrrole, mercaptans and aldehydes, oxidation and polymerization are the most important factors for the loss. Therefore, during the application and storage of MFs, it is difficult to maintain a stable concentration and flavor enhancing effect, especially in the thermal processing. It has become an important research direction for food scientists to analyze the mechanism of flavor generation and loss, and improve the controllability of flavor substances escape. In addition to high stability, MRIs can also reduce the bitterness of raw materials and increase the umami taste. It also improves the body's perception of salt, which helps reduce salt intake in daily life. But the mechanism of saltiness enhancement of vegetable protein enzymatic hydrolysates warrants future studies (10).

## Conclusion

The MRPs not only alter food properties (stability, flavor, color, etc.), but also enhance the functional properties (anti-oxidant, anti-microbial, and anti-browning) of amino acids, peptides and proteins. MRPs have been used as flavor enhancers because of good mouthfeel and umami. Their flavor characteristics could be pleasant floral, nutty, caramel, attractive meat, or even spicy. E-nose, E-tongue, and GC-MS have become the most representative tools to detect flavor components, helping researchers to obtain comprehensive flavor profile. MF depends largely on the proteins, reducing sugars, and conditions (time, temperature, pH, vacuum degree, etc.). After proteins and sugars obtained from different sources react, MRPs of different flavor are obtained. Meanwhile, under different enzymatic hydrolysis conditions, proteins are enzymolyzed into different peptide fragments. In MR, since the open chain concentration of sugars and active form of amino reactants depend on pH, each reaction and reactant has a different sensitivity to temperature and affects the reaction differently.

Except flavor, MR currently has many limitations in terms of the difficulty in controlling the reaction degree, and the instability of the final product. Undesirable substances, which could reduce the flavor and nutrition, might be formed during the reaction. To address these issues, MRI has become a new research focus and has contributed to improving stability and flavor, nevertheless, the generation and mechanism of MRPs still need to be further exploited.

## Author contributions

SL, GM, and LW designed the topic. LG and SL prepared the manuscript. HP, XL, and TZ prepared the figures. SL, GM, TZ, and HS reviewed and revised the manuscript. All authors contributed to the article and approved the submitted version.

## Funding

This work was funded by Technology Project of Anhui Province 201903a06020024, Technology Project of Anhui Province 202203a06020029, Eight Major Industrial Chain Strengthening and Chain Reinforcing Projects 2021GJ010, Science and Technology Plan Project of Huangshan City 2020KN-04, and Key Research and Development Projects from Anhui Province 202104a06020013.

## Conflict of interest

The authors declare that the research was conducted in the absence of any commercial or financial relationships that could be construed as a potential conflict of interest.

## Publisher's note

All claims expressed in this article are solely those of the authors and do not necessarily represent those of their affiliated organizations, or those of the publisher, the editors and the reviewers. Any product that may be evaluated in this article, or claim that may be made by its manufacturer, is not guaranteed or endorsed by the publisher.
